# BioTouch: Reliable Re-Authentication via Finger Bio-Capacitance and Touching Behavior †

**DOI:** 10.3390/s22093583

**Published:** 2022-05-08

**Authors:** Chong Zhang, Songfan Li, Yihang Song, Qianhe Meng, Li Lu, Mengshu Hou

**Affiliations:** School of Computer Science and Engineering, University of Electronic Science and Technology of China, Qingshuihe Campus, Chengdu 611731, China; zhangchong@std.uestc.edu.cn (C.Z.); sfli@std.uestc.edu.cn (S.L.); songyihang@std.uestc.edu.cn (Y.S.); qianhe@std.uestc.edu.cn (Q.M.); mshou@uestc.edu.cn (M.H.)

**Keywords:** reliable, user-transparent, re-authentication, touching behavior, bio-capacitance

## Abstract

Re-authentication continuously checks to see if a user is authorized during a whole usage session, enhancing secrecy capabilities for computational devices, especially against insider attacks. However, it is challenging to design a reliable re-authentication scheme with accuracy, transparency and robustness. Specifically, the approaches of using biometric features (e.g., fingerprint, iris) are often accurate in identifying users but not transparent to them due to the need for user cooperation. On the other hand, while the approaches exploiting behavior features (e.g., touch-screen gesture, movement) are often transparent in use, their applications suffer from low accuracy and robustness as behavior information collected is subjective and may change frequently over different use situations and even user’s motion. In this paper, we propose BioTouch, a reliable re-authentication scheme that satisfies all the above requirements. First, BioTouch utilizes multiple features (finger capacitance and touching behavior) to identify the user for better accuracy. Second, BioTouch automatically works during user operation on capacitive-touch devices, achieving transparency without the need for manual assistance. Finally, by applying finger bio-capacitance, BioTouch is also robust to various conditions, as this feature is determined by the user’s physical characteristics and will not change by different user positions and motions. We implement BioTouch for proof-of-concept and conduct comprehensive evaluations. The results show that BioTouch can flag 98% of anomalous behaviors within ten touching operations and achieve up to 99.84% accuracy during usage.

## 1. Introduction

Versatile computer systems call for continuous security provided by re-authentication schemes, guaranteeing that the current user is authorized, thereby protecting the system against insider attacks after it is unlocked. Meanwhile, with the development of the Zero-Trust security model [1,2,3], re-authentication is seen as a more secure way to provide continuous user authentication and authorization for the system. Without the user re-authentication process, an attacker could access an account either through initial authentication (e.g., by stealing passwords [4], copy fingerprints [5,6]) or simply using an open account of an authorized user who leaves the device unlocked. Hence, a reliable re-authentication scheme is essential for highly secure computational devices and should satisfy the following three requirements.

- Transparent in use. Re-authentication has to collect users’ behaviors during the whole usage session [7,8,9]. Thus, the scheme should be unobservable to the user until abnormal behaviors have been identified, failing which the user will suffer from being interrupted frequently.
- Accurate. Re-authentication schemes should have a low False Alarm Rate (FAR). Otherwise, an authorized user may be flagged as anomalous frequently, which hinders a consecutive use of the device.
- Robust. Re-authentication schemes should be available for diverse scenarios in practice, where users may use the device in different emotions, movements and body actions.

However, no existing re-authentication scheme can meet all the above requirements to the best of our knowledge. As shown in Table 1, the first approach is to intuitively use a camera facing the user’s face so as to identify who is operating the device [10]. However, to continuously record facial information, it requires the user to maintain a certain posture, restrict user operations during usage and also be sensitive to ambient light [11]. Meanwhile, with privacy concerns, users may not like to have their camera always on. Finally, face recognition algorithms could also be susceptible to impersonation attacks, such as the 3D mask [12].

Furthermore, features for re-authentication which are based on voice [13] and gait [14,15,16] are also considered in existing works. However, those re-authentication schemes are not transparent to users because they require the user to cooperate with the scheme (e.g., valid only when walking or talking), which is difficult to popularize [23].

Recently, usage transparency re-authentications have been attracting us since none of them require user cooperation during the usage session. Basically, they exploit the behavior information collected from a user’s operations via a Human–Machine Interface (HMI) such as mouse and touchscreen as the user freely operates the device. Based on a trained database from legitimate users, such usage transparency schemes can silently authenticate users without interrupting their normal operations [17,18,19,20,21,22]. User information is to be transparently collected and compared with the legitimate database to verify the legitimacy of the current user. However, the undesired robustness has become a major issue in practical use. The behavior information collected from the interfaces is subjective and will change and differ from intended purposes, situations and even the user’s emotions, which leads to false alarms. While an increase of authentication period could mitigate this issue [17], the re-authentication process will be complicated and delayed significantly, thereby hardly detecting abnormal behaviors at the early stage of insider attacks.

In this paper, we propose BioTouch, a reliable re-authentication scheme that continuously verifies a user’s identity during the whole usage session. To satisfy the aforementioned requirements, we utilize dual user features (bio-capacitance and touching behavior) in re-authentication and gather their merits. First, we utilize bio-capacitance for robust re-authentication. Specifically, the bio-capacitance stands for the capability of the human body to store charges, which is proportional to the number of cells and size of cell mass in the body, thus different for everyone [24,25]. By deploying bio-capacitance, BioTouch is able to be robust for various conditions as it is fixed by the user’s body and will not change in different application scenarios. Next, we combine touching behavior with bio-capacitance to perform successive identification and improve the accuracy. Finally, BioTouch collects both user features through the HMI during touching operation and imperceptibly authenticates users during the whole duration of the device use, continuously verifies the user’s identity and locks the device if the user is judged as an attacker. It can effectively provide continuous security insurance for an unlocked device during usage and is also transparent to the user.

Nevertheless, to realize BioTouch, we have to address the following technical challenges.

First, it is difficult to combine different user features to enhance the re-authentication as they have heterogeneous characteristics. Specifically, the bio-capacitance is a static feature which depends on the biological nature of the user’s body [26]. However, touching behavior is a dynamic feature implied in the user’s operation process. How can we get both features via capacitive sensors simultaneously? We settle this issue by utilizing the trend of the measured capacitance value in the touching (Section 4). Generally, we observe that the measured capacitance value is determined by both bio-capacitance of the user and the distance between the finger and the sensor. Hereby, user behavior with finger movement could be gotten through learning the changing paradigm of capacitance.

Second, while the behavior feature can be extracted from the collected capacitance value, we observe that the value may vary for the same person over different touchscreen operations such as tap, long-press and swipe. To address this problem, we design an operation-independent algorithm (Section 5.1) to pre-process the sampled capacitance data before being exploited to authenticate the user. The basic idea is to convert different types of touchscreen operations to a simple tap operation, thereby facilitating subsequent re-authentication.

In the rest of the work, we build up a user-legal model to identify the user more precisely. The basic idea is to identify the user based on multiple touching operations during usage, which can eliminate misjudgments and further improve the accuracy. This model assesses the user’s legality in each touching operation and accumulates the results to generate a legitimacy score. The score increases with legal operations and decreases with illegal operations. Once the score drops below the threshold, BioTouch would automatically block the session until the next admissive authentication.

To demonstrate the feasibility of our design, we prototyped BioTouch for proof-of-concept using Commercial Off-The-Shelf (COTS) components, including an SX9310 [27] chip (bio-capacitance sensing) and an Msp430f2132 [28] microprocessor (data processing). Then, we evaluated the prototype based on the data collected from 50 volunteers. To further evaluate BioTouch in long-time use, we utilized the collected data set and conducted mass simulations on 100,000 samples of attackers and legitimate users. For each sample, the simulations were iterated up to 50,000,000 times. The results show an interesting result—that BioTouch has increasing accuracy during usage, can identify 98% of attackers within six seconds (10 user-operations) and can find out all of them within 11 s (18 user-operations). Encouragingly, for legitimate users, the misjudgment is only 0.321% in a 16-min usage period (1000 user-operations) and only 0.895% during a 2.6-h usage (10,000 user-operations).

The main contributions made in this work are as follows:

- We propose BioTouch, a novel scheme for reliable user re-authentication that provides continuous security insurance for the device by transparently verifying the legality of the current user in every on-screen operation. It makes up the security issue of attacker preventing after device unlock that cannot be achieved by traditional authentication schemes, and provides a new idea to improve the security of computational devices as well as Zero-Trust model based systems.
- Our system is transparent during usage, and can continuously check the user’s identity without interfering in their normal operations, thereby effectively ensuring the continuous security of the device and maintaining a good user experience.
- We designed a novel data extraction algorithm (as described in Section 5.1) that can effectively eliminate the impact of different touching operations on user behavior and ensure the robustness of BioTouch in practical applications.
- We construct a user-legitimate model to describe the user’s legitimacy much more accurately, which can further improve the accuracy of the re-authentication.

The remainder of the paper is organized as follows: In Section 2, we introduce state-of-the-art related works and provide a background for our design. Section 3 introduces our system overview, and Section 4 presents the method of feature combination in BioTouch. Section 5 and Section 6 introduce our method of user feature extraction and re-authentication in detail, respectively. Section 7 introduces our prototype implementation. Section 8 sets up the experiments and discusses the results. Section 9 discusses the external influences and future works of BioTouch. Finally, we provide a summary of our work in Section 10.

## 2. Related Works

According to the required user features, the state-of-art re-authentication works can be classified into two categories:

### 2.1. Behavior-Based User Re-Authentication

To avoid frequent interruptions to users, user features should be imperceptibly collected for identification without user assistance. Conventionally, behavior habits (e.g., movement, trail) during the user operation are commonly considered as the most appropriate features for re-authentication as they can be collected via the Human–Machine Interface (HMI). The behavior features, which come from body motions, contain the habit information of the user [29] which could help in identification. For example, keystrokes [30] can be utilized to identify users via typing; touch behaviors [31] can also be collected as the user’s identity. These works enable user features to be transparently collected during user operation through HMI, thus providing a good user experience without the need for user assistance. However, user-behavior-based authentication suffers from its low user identification accuracy because of the limited stability of user behavior features, which may put the device at risk increasingly.

### 2.2. Biometric-Based User Re-Authentication

Biometric features come from user body characteristics, which are good at robustness and will not change by different user postures, situations and emotions [32,33]. However, biometric features are static information, which has to be sensed under user assistance (e.g., touching the fingerprint panel). In re-authentication, the user’s biometric information needs to be collected frequently, which requires the user to frequently repeat specific operations. For example, keep facing the camera for continuous face recognition [10] or constantly talk [13] to assist the re-authentication progress. Despite the fact that those works may achieve high security in terms of device protecting, the frequent requirements of user assistance lead to poor user experience.

In summary, none of the state-of-art works can meet the aforementioned three requirements for reliable re-authentication scheme design. Hence, in this paper, we aim to propose a new scheme for reliable user re-authentication. It should be able to utilize multi-denominational features from the user to provide an accurate and robust user re-authentication. Besides, the feature collection and authentication progress should be transparent to the user to provide a good user experience.

## 3. BioTouch in a Nutshell

**Figure 1:**
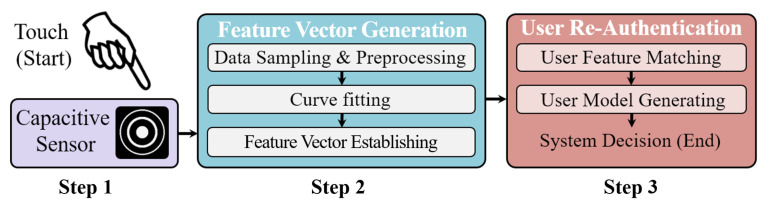
During touching operation, BioTouch transparently verifies the current user’s legitimacy through the following three steps: Combined user feature collection (Step 1, Section 4), user feature vector generation (Step 2, Section 5) and user re-authentication (Step 3, Section 6).

The target of BioTouch design is to provide continuous security insurance for computational devices during usage as they are already unlocked. As presented in Figure 1, the re-authentication progress on a BioTouch system is mainly made up of three steps, as follows:

- Step 1: Combined user feature collection. In the first step, BioTouch transparently collects both the bio-capacitance and touching behavior features from the user as they freely operate the device. The feature collection progress is transparent, and both the features are simultaneously collected via the sensor without attracting the user’s attention. The details are presented in Section 4.
- Step 2: Feature vector generation. In the second step, BioTouch generates a four-dimensional feature vector as the identity characteristic for the user based on the information collected in step 1. In this progress, BioTouch samples the sensor and pre-processes the sampled data to remove the impact from different types of touching operations. Then, BioTouch fits the curve and establishes the feature vector based on the coefficients of curve functions. The design details are presented in Section 5.
- Step 3: User re-authentication. In the final step, BioTouch continuously authenticates the user’s legitimacy by comparing the generated feature vector with the stored legitimate database. If it matches, the system judges the current user’s identity is legal. Otherwise, it is illegal. Based on multiple authentication results in the usage, BioTouch generates a user-legitimacy model with a user legal score to eliminate misjudgment and describe the user’s legitimacy more accurately. The score dynamically updates during the usage session, which increases if the result is legal and drops if the result is illegal. If the score drops below the legitimate threshold, BioTouch locks the device and forces the user to log out to avoid possible malicious access to the private data. The design details are presented in Section 6.

## 4. Combined Feature Collection

**Figure 2:**
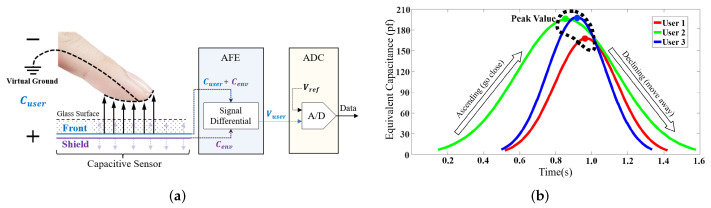
Using the capacitive sensors in a touchscreen, BioTouch continuously authenticates users according to their bio-capacitance during finger touching. (**a**) The collection of equivalent capacitance ($C_{user}$). (**b**) The change of equivalent capacitance ($C_{user}$) during touching.

In the first step, BioTouch transparently collects both the bio-capacitance and touching behavior features from the current as basis for the re-authentication. During the touching operation, the bio-capacitance and touching behavior features of the user are extracted through the peak value and slew rate of the collected capacitance value, respectively. As shown in Figure 2a, when the user’s finger approaches the capacitive screen, an equivalent capacitor ($C_{user}$) is created between the finger and the sensor under the screen. The capacitive sensor consists of two parts, front and shield. The front collects the user’s bio-capacitance, but is vulnerable to environmental influences. Hence, the Analog Front End (AEF) utilizes the shield to sense the environment ($C_{env}$) and eliminates environmental interference through signal differentiation. The AEF also generates voltage signals ($V_{user}$) based on the sensed user capacitance ($C_{user}$) so that the Analog-to-Digital Converter (ADC) can output the collected capacitance data in digital form based on the reference voltage ($V_{ref}$).

The collected capacitance value is determined by the user’s bio-capacitance as well as the distance between the finger and the sensor, which increases as the finger approaches and decreases as the finger moves away. Given that different users have unique bio-capacitance values, BioTouch leverages the peak value of the measured capacitance curve to distinguish users, where the distance between the finger and sensor is minimized and equal to the thickness of the glass. Further, we also exploit the slew rate of the capacitance curve (the rate of ascending and declining) to represent the behavior of the user as an additional dimension to improve the accuracy. Specifically, users have unique behavior features in on-screen operations (finger moving speed) according to their habits. The finger movement speed results in a change to the distance and finally results in the change of sensed capacitance value, as shown in Figure 2b.

For better understanding, we use mathematical description to explain how the above-mentioned combination of bio-capacitance and touching behavior can be used to identify different users accurately. First, when a user’s finger approaches the screen, the electric charges will accumulate on the surface of the finger due to the electric field, and the quantity ($Q_{user}$) of the charge is proportional to the user’s bio-capacitance ($C_{user}$) [24]:

(1) $$Q_{user}\propto C_{user}$$

According to the capacitance function, we know that the capacitance value is inversely proportional to the distance between two conductors. Besides, the tread of distance change is related to the user habit during the touching. Let the distance between the finger and the sensor be $D_{user}\left(t\right)$; then, we can get:

(2) $$C_{measure}\left(t\right)\propto \frac{Q_{user}}{D_{user}\left(t\right)}\propto \frac{C_{user}}{D_{user}\left(t\right)}$$

In the equation, $C_{measure}\left(t\right)$ is a function with independent variable *t* (the time of the touching) to represent the measured capacitance by the sensor. Hence, it contains the user feature of bio-capacitance ($C_{user}$) as well as touching behavior ($D_{user}\left(t\right)$). By this, we achieve feature combination for user re-authentication.

## 5. Feature Vector Generating

### 5.1. Data Sampling and Pre-Processing

The first step in user feature extraction is data sampling. In this step, the system periodically reads the sensor to obtain capacitance data simultaneously as the user touches the screen. Indeed, as we described in Section 3, the obtained capacitance data contains both bio-capacitance and touching behavior information of the user, which can be exploited to identify different users. However, holding periods such as long-term finger pressing and swiping may occur due to certain touching gestures, changing the sensed capacitance value and then leading to error in further identification.

**Figure 3:**
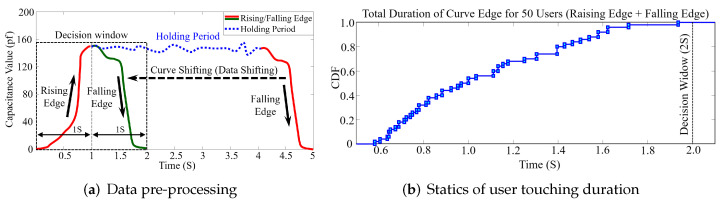
We preprocess the data to eliminate the impact of different touch operations based on an appropriate time window according to user habits. We extract the rising and falling part of the sensed data and remove the holding period to avoid the influences from different touch operations (**a**). The Cumulative Distribution Function (CDF) statistics of user habit, where all 50 users complete the tap motion within 2 s (**b**).

To eliminate the impact of different operations on identification, we designed a novel algorithm to pre-process the data simultaneously during sampling. The algorithm is designed to transform all types of touching operations into simple tap operations which only contain the finger approaching and moving away moments. Specifically, the algorithm distinguishes and eliminates the holding period; thereby, only the ascending and descending segments of capacitance value are reserved, which are related to user behaviors, as shown in Figure 3a. The trends of sensed capacitance value are simultaneously detected during data collection. If the value does not change significantly over a period of time (decision window), it will be defined as a holding period and deleted. To set the appropriate length of the decision window, we analyzed the tapping habits of 50 users, and the statistical results are shown in Figure 3b. We observe that the touching time duration of all users (without holding period) is within 2 s. Hence, we set 1 s as the length of the decision window to obtain each rising and falling edge in the curve (two seconds in total). The logic of the algorithm is shown in Algorithm 1.
**Algorithm 1** Capacitance Extraction1:⊳Variableinitialization2:NewFileString3:NewArrayP[length][3]4:time←0,value←0,trend50←0,trendflag←05:⊳Dataprocessing6:**for**String.readLine()!=null **do**7:   timenext←String[0]8:   valuenext←String[1]9:   **if** |timenext−time|>timethreshold **then**10:     Continue11:   **else**12:     trendflag←113:     P←timenext,valuenext,trendflag14:     Continue15:   **end if**16:   **if** valuenext−value>0andtrend50==0 **then**17:     trendflag=018:     P←timenext,valuenext,trendflag19:     Continue20:   **end if**21:**end for**22:⊳Calculatethevalue23:**for** fori←0tolength[P]**do**24:   **if** flag←P[i][3]==1 **then**25:     count←count+126:     time←time+P[i][0]27:     value←value+P[i][1]28:   **end if**29:   timeavg←P[i][0]/count30:   valueavg←P[i][1]/count31:   Quick−Sort(P)32:**end for**

### 5.2. Curve Fitting

**Figure 4:**
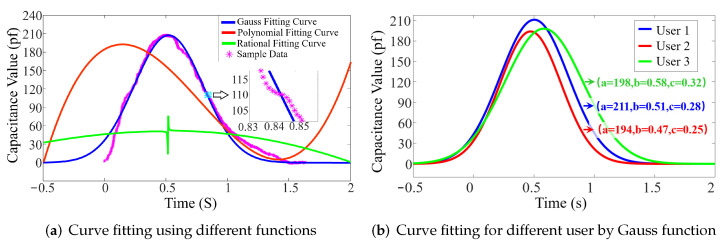
(**a**) Curve fitting base on different functions. (**b**) Gauss function fitting for three users.

After data sampling and pre-processing, BioTouch gets the discrete capacitance value. These discrete points contain both the bio-capacitance and touching behavior information of the user. To achieve user identification, we need to extract user features from thousands of sampling points. Therefore, we perform curve fitting on the collected data so as to use the function parameters of the fitted curve to represent different users. We tested various fitting methods, including Gaussian [34,35,36], Polynomial [37,38,39] and Rational numbers [40], and found that the Gaussian curve matches the collected data most (Figure 4a). Hence, BioTouch uses a Gaussian function (Equation (3)) to fit the curve for user identification. The Gaussian function has three coefficients: The peak value of the curve (a), the abscissa value in the center of the curve (b), and the half-width of the curve (a). BioTouch utilizes these three coefficients and builds up a 3D feature vector for the user. We can see there are obvious differences between different users in Figure 4b. The expression of the Gauss equation is as follows:

(3) $$f\left(x\right)=ae^{-\frac{{\left(x-b\right)}^{2}}{2c^{2}}}$$

### 5.3. Feature Vector Establishing

Further, we need to solve Equation (3) to get the value of Gauss curve coefficients, which meanwhile minimize the variance between the fitted curve and the sensed data. First, we use logarithms and simplify the function as follows:

(4) $$ln\left(y_{i}\right)=\left\{ln\left(a\right)-\frac{b^{2}}{2c^{2}}\right\}+\frac{2x_{i}b}{2c^{2}}-\frac{x_{i}^{2}}{2c^{2}}$$

Next, let $ln\left(y_{i}\right)=Z_{i}$, $ln\left(a\right)-\frac{b^{2}}{2c^{2}}=b_{0}$, $\frac{2b}{2c^{2}}=b_{1}$, $-\frac{1}{2c^{2}}=b_{2}$. When these three equations are substituted into Equation (4), we obtain:

(5) $$Z_{i}=b_{0}+b_{1}x_{i}+b_{2}x_{i}^{2}=\left(\;1\;\;x_{i}\;\;x_{i}^{2}\;\right)\left[\begin{array}{c} b_{0}\\ b_{1}\\ b_{2} \end{array}\right]$$

Next, we define the sampled capacitance value from the user’s single operation as:

(6) $$\left[X,Y\right]=\left[\left(x_{1},y_{1}\right),\left(x_{2},y_{2}\right),\;\cdots \left(x_{i},y_{i}\right),\;\cdots \right]$$

where $x_{i}$ is the sampling time of the sensed bio-capacitance and $y_{i}$ is the sampled value (*i* means the *i*-th sampling point). We import all the sampled data into Equation (5) and get the following parameter matrix:

(7) $$\left[\begin{array}{c} Z_{1}\\ Z_{2}\\ \vdots \\ Z_{n} \end{array}\right]=\left[\begin{array}{ccc} 1 & x_{1} & x_{1}^{2}\\ 1 & x_{2} & x_{2}^{2}\\ \vdots & \vdots & \vdots \\ 1 & x_{n} & x_{n}^{2} \end{array}\right]\left[\begin{array}{c} b_{0}\\ b_{1}\\ b_{2} \end{array}\right]+\left[\begin{array}{c} \varepsilon_{1}\\ \varepsilon_{2}\\ \vdots \\ \varepsilon_{n} \end{array}\right]$$

This matrix can be further simplified as:

(8) $$Z_{n\times 1}=X_{n\times 3}B_{3\times 1}+E_{n\times 1}$$

To minimize the total variance of the calculated results, we get matrix *B* according to the principle of least squares:

(9) $$B={\left(X^{T}X\right)}^{-1}X^{T}Z=\left[\begin{array}{c} b_{0}\\ b_{1}\\ b_{2} \end{array}\right]=\left[\begin{array}{c} ln\left(a\right)-\frac{b^{2}}{2c^{2}}\\ \frac{b}{c^{2}}\\ -\frac{1}{2c^{2}} \end{array}\right]$$

By solving the above equation, we can obtain the optimal values of the three main parameters $\left(a,b,c\right)$ for user features as follows:

(10) $$a=e^{b_{0}-\frac{b_{1}^{2}}{2b_{2}^{2}}}$$

(11) $$b=-\frac{b_{1}}{2b_{2}}$$

(12) $$c=\sqrt{\frac{-1}{2b_{2}}}$$

Next, we add the box plot of the collected capacitance data as an additional parameter to further enhance the integrity of user features. Specifically, the box plot is a method which leverages the quantiles of groups of numerical data to graphically depict them [41,42,43,44,45], which can effectively reflect the overall changes and distribution of the entire data set. The typical quantiles are 25% quantile, 50% quantile (median value), 75% quantile, upper boundary (maximum value) and lower boundary (minimum value) in the data set.

**Figure 5:**
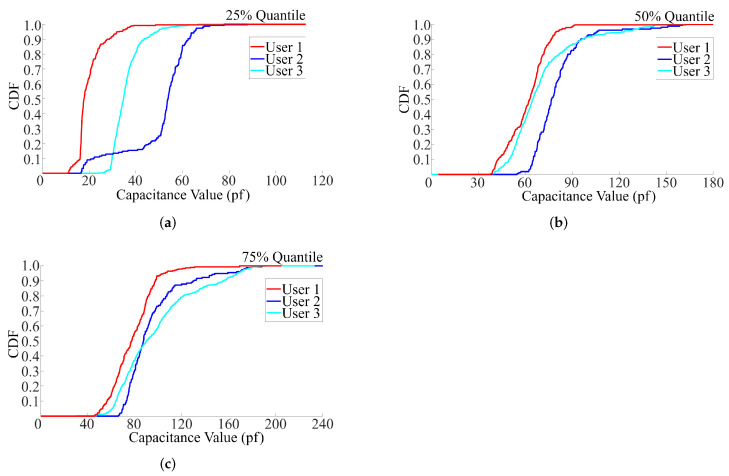
CDF of 25% (**a**), 50% (**b**) and 75% (**c**) quantiles based box plot of the collected data in touching.

Based on the above quantities, we tested the collected data and found that the 25% quantile-based box plot has the best discrimination among different users, as shown in Figure 5. Therefore, we added the 25% quantile of the collected capacitance to user features and combined it with curve parameters (*a*, *b*, *c*) to build up a four-dimension feature vector for user identification.

## 6. User Re-Authentication

### 6.1. Feature Matching

**Figure 6:**
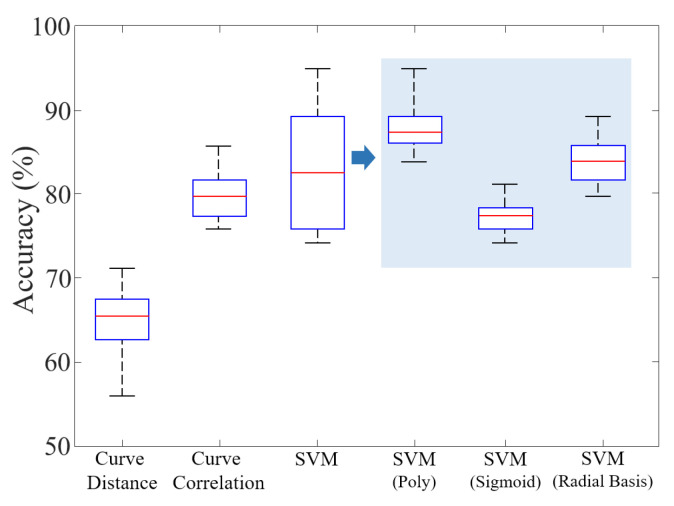
Classification accuracy of a single user touch operation under different methods. It can be clearly seen that the SVM with poly-function does the best, which we choose in BioTouch for user feature matching.

In this step, BioTouch compares the generated feature vector with the pre-stored data to verify the current user’s legitimacy. To find a suitable algorithm for user authentication in BioTouch, we tested familiar classification and matching algorithms, including curve distance, curve correlation, and Support-Vector-Machine (SVM). As shown in Figure 6, we found that SVM performed best. The SVM is a supervised learning model which has associated learning algorithms to analyze the data for classification. Specifically, a support vector machine constructs a hyperplane or set of hyperplanes in a high-dimensional space, which can be used for classification [46]. Intuitively, a good separation is achieved by the hyperplane that has the largest distance to the nearest training-data point of any class since, in general, the larger the distance, the lower the generalization error of the classifier [47]. Employing different kernel functions, SVM achieves different performances. We conducted corresponding experiments and found that the polynomial kernel achieved the best results. Hence, we deploy SVM with polynomial kernel in BioTouch for user feature matching. The equations of the mentioned kernel functions are shown below:

$\mathrm{Polyomial}\;\mathrm{Kernel}:\;K\left\{x,y\right\}={\left({\mathrm{ax}}^{T}y+c\right)}^{d}$.

$\mathrm{Radial}\;\mathrm{Basis}\;\mathrm{Kernel}:\;K\left\{x,y\right\}=\exp\left(-a{∥x-y∥}^{2}\right)$.

$\mathrm{Sigmoid}\;\mathrm{Kernel}:\;K\left\{x,y\right\}=\tanh\left({\mathrm{ax}}^{T}+c\right)$.

In the above function, x is the abscissa value, y is the ordinate value, T is the matrix transpose, exp is the exponential of the natural constant e and a and c are constants.

### 6.2. User Model Generating

To realize the re-authentication, we build a user model to describe the user’s legitimacy more precisely. This model transforms multiple identification results obtained within a certain period into a user-legitimacy score. This score is closely related to the legitimacy of the user’s identity, which decreases when an illegal user operates on the screen, and vice versa. This model works imperceptibly in the background, thereby achieving transparency for the user. When the score drops below the threshold, BioTouch make the user log out because the user is possibly unauthorized. The parameters of the algorithm should be carefully selected so as to effectively detect authorized access while lowering the false recognition rate, as shown in Algorithm 2.
**Algorithm 2** User-legitimacy ModuleInput: Authentication result of each finger-touching operation.Output: User’s legitimacy score and system decision (Keep user login or kick user out).1:Start2:Score=1003:Operationtimes:i=04:**for** Score≥0 **do**5:   i++6:   **if** Authentication result[i]=illegal **then**7:     Score=Score−plentypoints*X*8:     **if** (i≥2) **and** (Authentication result[i−1]=illegal) **then**9:        Score=Score−extra plenty points *E*10:     **end if**11:   **else**12:     Score=Score+correction points *Y*13:   **end if**14:   **if** Score>upper bound *S* **then**15:     Score=upper bound *S*16:   **end if**17:   **if** Score<0(Threshold) **then**18:     Tick the user out19:   **end if**20:**end for**

This algorithm has four parameters: Penalty points (x) for illegal operations, correction points (y) for legal operations, extra penalty points (e) for continuous illegal operations, and an upper bound (s). After successfully logging in, the user will get an initial score of 100. The score decreases by x points every time an unauthorized user is detected or increases by y points every time an authorized user is detected. If the unauthorized user is continuously detected, the algorithm deducts extra penalty points by e points. After the *i*th user operation, the expectation of user score can be expressed as

(13) $$S\left(i\right)=100-ipx+i\left(1-p\right)y-\left(i-1\right)p^{2}e$$

where S(i) is the expectation value of the user’s score after the *i*-th user operation, and *p* is the average probability for the user to be judged unauthorized each time. If S(i) drops below the threshold (0), BioTouch makes the current user log out to ensure system security.

## 7. Implementation

### 7.1. Hardware Implementation

To validate the feasibility and performance of BioTouch, we build a hardware prototype with COTS components. However, we cannot directly deploy BioTouch on existing capacitive touchscreens since they are designed for precise touching coordinate detection but their accuracy of capacitance measurement does not satisfy the requirement of BioTouch. In detail, to achieve precise coordinate detection while lowering the manufacture cost, most capacitive touchscreens are based on projected capacitive touch (PCT) technologies [48,49,50,51], which are comprised of millions of micro-capacitors in a mixed array [52,53], and only use a single threshold analog-to-digital converter (ADC) to sense the bio-capacitance. Hence, it can precisely detect the coordinate where the finger touches, but fails to measure the specific capacitance value. Thus, we need to come up with a new implementation of touch screen with sensitive capacitance sensor and high-resolution ADC to deploy BioTouch.

Logically, such a screen is not difficult to customize, as we only need to add a high-resolution capacitive sensor under a traditional displaying screen. However, in fact, the BioTouch system does not require the display function, so we use COTS components for system implementation.

**Figure 7:**
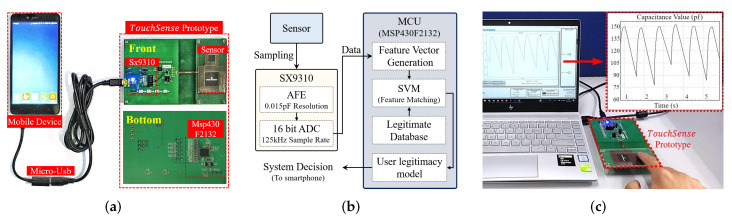
BioTouch contains a novel designed PCB to control the device. We can also use a laptop to monitor the working states of the prototype, where the sensed capacitance value is shown in the window in real time. (**a**) BioTouch prototype. (**b**) Prototype block digram. (**c**) Working status monitoring of BioTouch.

We chose a high-resolution sensor chip (SX9310 [27]) for bio-capacitance sensing and an Ultra-Low-Power MCU (Msp430f2132) [28] for data processing in user re-authentication. The system prototype and block diagram are shown in Figure 7a,b, respectively. When the user approaches her/his finger to the sensor, the SX9310 chip samples the sensor by its Analog Front-End (AEF) and digitalizes the data by a 16 bit ADC. Then, the MCU generates feature vectors based on the collected data and builds up the re-authentication by comparing the generated features with the stored legitimate database by the Support Vector Machine (SVM). Based on the re-authentication results, the MCU builds up user legitimacy model and gives out the final decision for the smartphone (whether to lock the device and force users to log out). We can also use a laptop to monitor the working status of BioTouch, as shown in Figure 7c.

We list the parameters in our prototype in Table 2. Specifically, our system adopts an event driven work mode to save power and extend battery life (the detail is described in Section 8.8), where the scan period in standby mode and the sample rate in work mode is 30 ms and 125 kHz, respectively. Next, to smooth the sensed value, we set up a filter to get the average value in every 16 samples, which can effectively eliminate the interference from signal jitters. In order to be compatible with more users, we set the range to the maximum value (7.5 pF) and set the signal gain at 32 times in the program to better distinguish different users. By this, the set sensing range and resolution are 240 pF and 0.015 pF, respectively, where the resolution is 1/16,384 in the value of the sensing range in the sensor setting.

### 7.2. User Model Parameter Setting

BioTouch has four parameters (x,y,s,e) in the user model (Section 5.2), which affects the performance of re-authentication. Specifically, re-authentication systems should quickly flag out attackers and avoid misjudgment for legitimate users as possible. To find the optimal parameter value, we made the following calculations. First, assuming that the average probability of a user to be judged as illegal in each operation is *p*, after the *i*-th operation, the expected score S(i) can be expressed as:

(14) $$\begin{array}{c} S\left(i\right)=\mathrm{Max}\left\{100-ixp+iy\left(1-p\right)-\left(i-1\right)ep^{2},s\right\} \end{array}$$

To improve security and user experience, we need to find the optimal parameters to quickly deduct the score of attackers ($S_{Attacker}\left(i\right)$) and force them to log out with less operations (*i*) and raise the score of legitimate users ($S_{Legal}\left(i\right)$) to the upper bound *s* to create space for fault tolerance. We set operable times (*i*) of attackers and legitimate users as a performance indicator. The operable time means the total touching operations for a user who continuously operates until logged out by BioTouch due to the score dropping below the threshold and being defined as an intruder. Hence, BioTouch should offer less operable times for attackers and more for legitimate users. We tested 50 users and found that the probability is 83.88% for attackers to be considered as illegal in a single touching operation ($p_{att}=0.8388$) and only 9.36% for legitimate users ($p_{leg}=0.0936$). Based on Equation (14), the expected operable time for attackers and legitimate users can be expressed as Equations (15) and (16), respectively.

(15) $$i_{attacker}=\left\lceil \frac{100+p_{att}^{2}e}{p_{att}x-\left(1-p_{att}\right)y+p_{att}^{4}e}\right\rceil$$

(16) $$\begin{array}{c} I_{legal}=\left\lceil \frac{100+p_{leg}^{2}e}{p_{leg}x-\left(1-p_{leg}\right)y+p_{leg}^{4}e}\right\rceil \\ +\frac{1}{\sum_{1}^{\infty }\left(\frac{{\left(1-p_{leg}\right)}^{i}\cdot p_{leg}^{\left\lceil \frac{s+\left(i+1\right)e+iy}{x+e}\right\rceil }}{\left\lceil \frac{s+\left(i+1\right)e+iy}{x+e}\right\rceil +i}\right)} \end{array}$$

By solving the above functions, we can get the value of these four parameters (x,y,s,e) as shown below:

Penaltypoints:x= 25; if a user has an illegal operation, the score will be reduced by 25 points.

Correctionpoints:y= 67; if a user has a legal operation, the score will increase by 67 points.

ExtraPenaltypoints:e= 40; if the user makes continuous illegal operations, the score will be deducted by an extra 25 points.

UpperBound:s= 568; the maximum score for legitimate users, which keeps their score away from the logout threshold. This parameter is a fault-tolerant mechanism for legitimate users, allowing them to make some mistakes.

**Figure 8:**
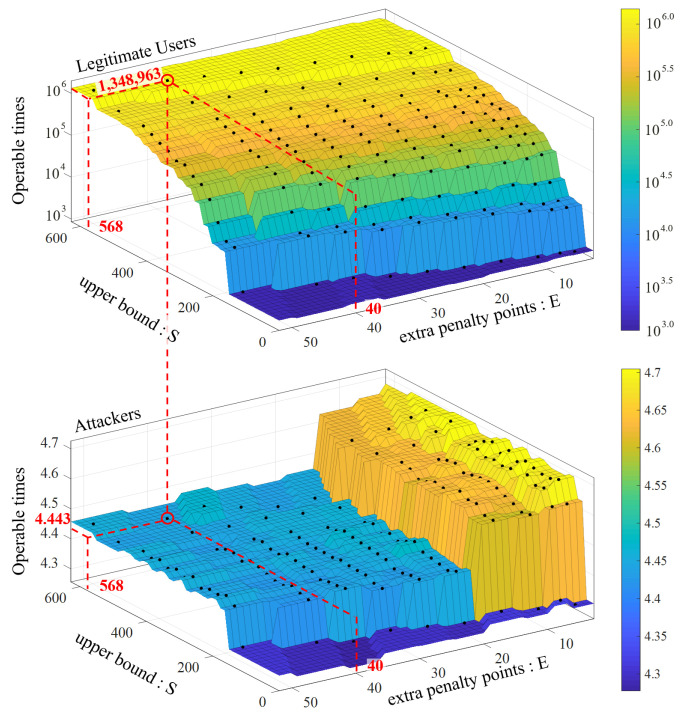
Average operable times of attackers and legitimate users with the change of the upper bound S and extra penalty points E. With a configuration of S = 568 and E = 40, legitimate users can on average operate the device 1,348,963 times before logout, but attackers can only operate the device 4.443 times before being flagged out. In this figure, other parameters remain unchanged as penalty points X = 25 and correction points Y = 67.

In those four parameters, the upper bound (*s*) and extra penalty points (*e*) affect the results most. Specifically, the attacker’s operations are more likely to be identified as illegal thereby lead to deducting extra penalty points (*e*). Besides, the space between the upper bound (*s*) and the threshold (0) provides a fault-tolerant space, as legitimate users may be occasionally identified as attackers due to misjudgment. In order to demonstrate this feature, we simulated the average operable (*i*) times for 100,000 samples of attackers and legitimate users with different values of upper bound (*s*) and extra penalty points (*e*); the result is shown in Figure 8.

With this configuration, attackers can be marked and logged out by BioTouch after 4.443 touch operations on average, which ensures device security. However, for legitimate users, they can on average operate the device 1,348,963 times before they are wrongly identified as an attacker, thus ensuring a good user experience.

## 8. Evaluation Results

To evaluate the feasibility of BioTouch, we test the prototype with 50 users, including tapping, long-pressing and swiping operations. Further, we conduct simulations based on the collected data to evaluate our system in long-time use.

### 8.1. Touching Detection Rate

We first evaluated the touching detecting rate of our prototype in finger touching. We assign users to repeat a touching operation (tapping, long-pressing and swiping) for one minute and compare the number of detected operations with the actual number. The experiment results show that 98.9% of touching operations can be successfully detected. As an example, the test results of ten users are shown in Table 3. In the above touching operations, tapping has a negligible duration of holding period, much shorter than the duration in long-pressing and swiping, thus causing completely different trends in sensed capacitance value. With the solution introduced in Section 5.1, BioTouch achieves a good detection rate in diverse touching operations.

### 8.2. User Differentiation Rate

To find the boundary for Biotouch to differentiate different users, we designed a test to examine the classification accuracy of our system under different differences of user features. Specifically, Biotouch builds up the four-dimensional feature vector (as described in Section 5) for each user and then distinguishes by the SVM classifier (as described in Section 6). Therefore, the difference of coefficients in the feature vector will greatly affect the classification accuracy. To control variables and set up the test, we first generated 2000 virtual users based on collected data from 50 real users. Among the virtual users, 500 samples have differences only on one coefficient in their feature vectors, 500 samples have differences on two coefficients, 500 samples have three coefficient differences and the last 500 sample users have differences in all the four coefficients on their feature vectors.

**Figure 9:**
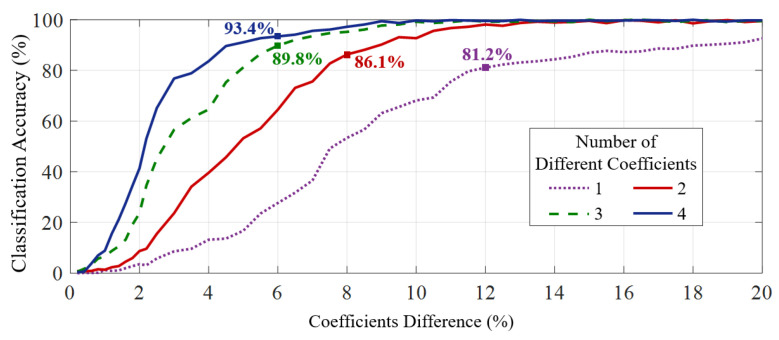
User classification accuracy under different degrees of feature differences.

The results of classification accuracy are presented in Figure 9. We can clearly find that the difference in user features has a great influence on user differentiate rate. Specifically, with 8% difference in two coefficients, the system can achieve 86.1% classification accuracy for different users. Further, with 6% difference in three and four of the coefficients, the classification accuracy increases to 89.8% and 93.4%, respectively. With difference in more coefficients, the classification accuracy improves significantly as feature differences on rich dimensional make it easier for the SVM to distinguish different users. In practical scenarios, benefit from differences in physical characteristics and usage habits, most users have significant differences in two or more coefficients, and BioTouch can distinguish them effectively.

### 8.3. Confusion Matrix of Feature Vector for 50 Users

**Figure 10:**
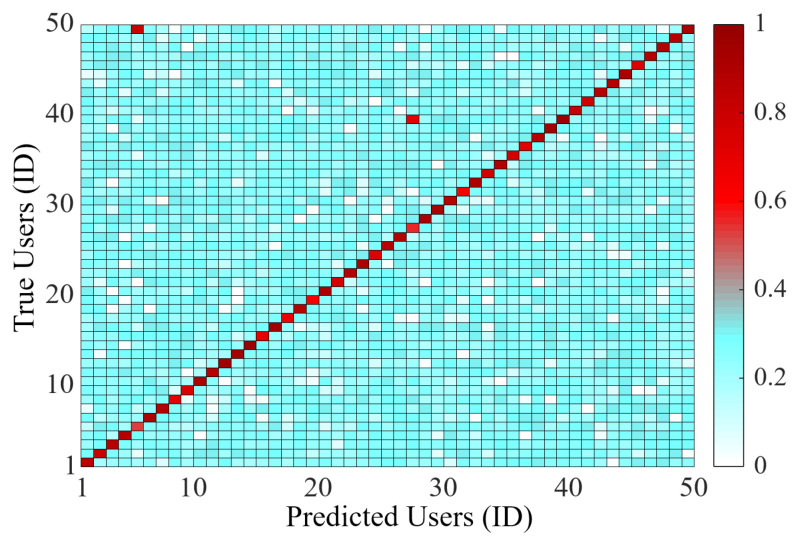
Confusion matrix for 50 Users. Among the 50 users, only two users were mispredicted, and the overall accuracy is up to 96%.

To evaluate the overall performance of BioTouch, we build up the confusion matrix [54] based on collected data from 50 users, as shown in Figure 10. In this figure, the abscissa value is the predicted users based on the data set, and the ordinate value is the ID of real users. Besides, the color depth in the figure means the confidence of system prediction. The results show that the system only has two wrong identification results among all 50 users (accuracy = 96%).

### 8.4. Re-Authentication Performance under 100,000 Samples of Legitimate Users and Attackers

**Figure 11:**
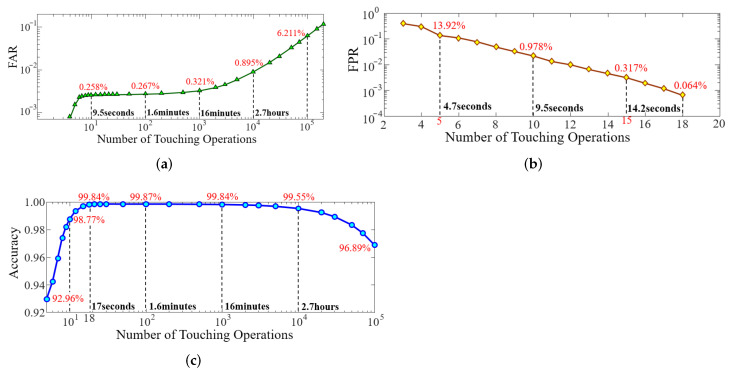
System performance of BioTouch during usage. (**a**) False Alarm Rate (FAR) for legitimate users. (**b**) False Positive Rate (FPR) for attackers. (**c**) System accuracy of BioTouch.

To further evaluate the overall performance of BioTouch, we conducted mass simulations on 100,000 samples of attackers and legitimate users based on the collected data. The False Alarm Rate (FAR) and False Positive Rate (FPR) and accuracy of BioTouch at different number of touching operations are shown in Figure 11.

The False Alarm Rate (FAR) (Figure 11a) shows the rate of legitimate users falsely alarmed and forced to log out by BioTouch. The lower the FAR, the fewer legitimate users are misjudged and logged out by BioTouch. From the simulation results, we can see that only 0.258% of the legitimate users are falsely misjudged by BioTouch in ten operations (9.5 s on average), and the value is below 0.27% after 100 operations. As user re-authentication is a repetitive process, the number of misjudged legitimate users accumulates during usage. Hence, the FAR increases slowly with more operations. For most usage scenarios (100∼1000 operations, tens of minutes), the FAR is less than 0.321%. For continuous long-time usage (10,000 operations, 2.64 h on average), the FAR is also lower than 0.9%.

The False Positive Rate (FPR) (Figure 11b) shows the rate of attackers who were missed and left in the system, which represents the security performance of BioTouch (lower is better). The results show that only 2.19% of attackers are not identified by BioTouch after 10 operations (9.5 s on average), and all attackers are forced to log out after 18 operations (17 s on average). Hence, BioTouch can identify 97.8% of attackers within 10 s and find out all of them within 17 s.

The accuracy (Figure 11c) reflects the overall performance of BioTouch. The results demonstrate that the system accuracy changes as the number of user operations increases. Specifically, in the first 18 operations, the accuracy increases and then reaches the peak (99.87%) between 18 and 1000 operations. However, the accuracy begins to drop after 1000 operations, which is 99.84% in 1000 operations and 99.55% in 10,000 operations. The reason stems from the change of FAR and FPR. In the first 18 operations, the FPR drops rapidly and the FAR keeps close to 0, thus making the accuracy increase. Conversely, after 1000 operations, FAR increases slowly due to the accumulation of misjudgment and finally results in the gradual drop of accuracy. According to statistics [55], in daily life, users like to use the device for tens of seconds to tens of minutes after unlocking the device, which equates to 20 to 1000 touching operations in a single usage. In this case, BioTouch provides not less than 99.84% accuracy and no more than 0.16% FIR.

### 8.5. Motion Influence

**Figure 12:**
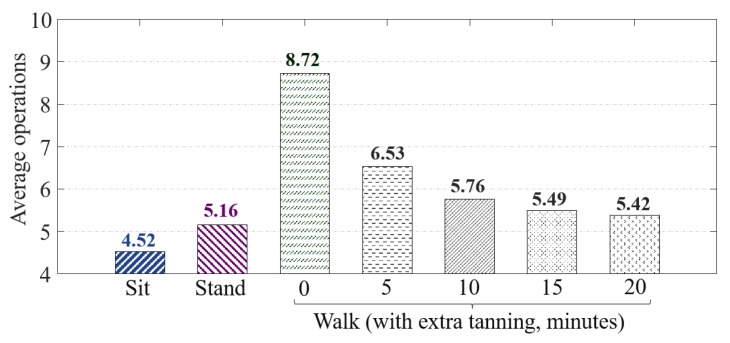
The average operation required for our system to log out attackers in the scenario of user sitting, standing and walking.

In order to evaluate the system performance under different motion influences, we tested the time cost of our prototype to distinguish attackers in the scenario of a user walking, standing and sitting. We selected ten users for this test and collected the data on 60 touches from each user in the sitting posture. In the test, we took turns to select a user as the device owner and let the other nine users as attackers operate the device and record their operation times before they are identified and logged out by the system. To eliminate the randomness, we repeat each test 20 times and take the average value. The results are presented in the Figure 12.

The results demonstrate that in the sitting and standing states, the system can quickly detect and log out the attacker within a few operations, which only requires 4.52 and 5.16 operations on average, respectively. However, in the walk state, the system performance decreases, where an attacker can operate the device at 8.72 times before being detected by the system (without additional training), which is 93% higher than that in the sitting states.

To address this issue, we took additional data trained on the system by collecting the data during user touching in walking. Then, we re-performed the test after additional training for 5 min, 10 min, 15 min and 20 min. In such training, a user can perform 50 to 65 touch operations in a minute to record the data and enhance their data set in the walking state. The test results show that the system performance significantly improved after only 10 min of training, where the attackers can be found and logged out within six operations. In this state, the attacker cannot even type a long word. Therefore, even in the waking state, our system can effectively protect the safety of the device, which only needs to collect the touch operation of the owner as the legitimate data set for 10 min during walking.

### 8.6. Time Cost to Identify a User

**Figure 13:**
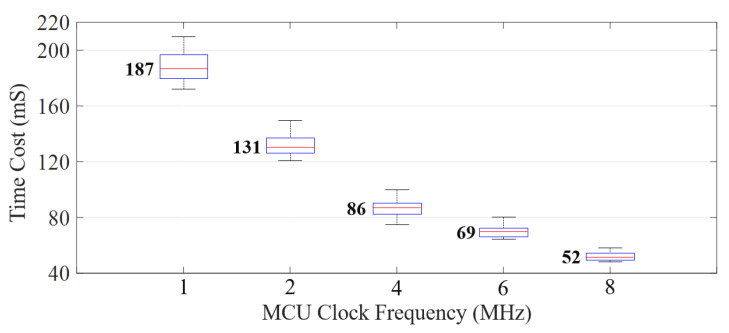
The time cost to identify a user under different clock frequency of the microcontroller (MCU).

We tested the time cost for our prototype to authenticate a user in a single touch, as shown in Figure 13. We set different clock frequencies to run the microcontroller (MCU). We wrote an embedded program to record the time cost from when the user completes touching to when the authentication results are output. The results demonstrate that our system works fast in user authentication. With 1 MHz clock driven, the system can give out the results within 200 ms after the user touches. Moreover, the time cost can be further reduced to 52 ms if we improve the clock frequency to 8 MHz.

### 8.7. Long-Time Usage Simulation for 100,000 Samples of Legitimate Users and Attackers

**Figure 14:**
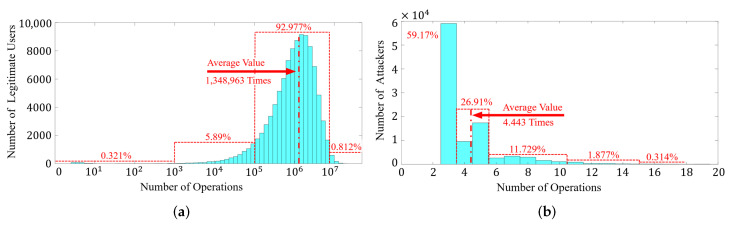
Most attackers are flagged out within six operations, while only a small part of legitimate users are misjudged within 100,000 times of continuous operations. (**a**) Histogram of operable times for legitimate users. (**b**) Histogram of operable times for attackers.

We simulated the operable times for legitimate users and attackers based on 100,000 samples of each, as shown in Figure 14. We can see that 92.977% of the legitimate users can operate the device for 100,000 to 10,000,000 times before being forced to log out by BioTouch due to misjudgement (Figure 14a). The average value is 1,348,963 times, which means the legitimate users can continuously operate the device for 356 h on average before the first misjudgment, which is far beyond required usage for most ordinary users. If we consider that users are likely to operate the device for 15 min (945 operations) each time after unlocking. In this scenario, less than 0.321% of the legitimate users will be misjudged by BioTouch.

However, for attackers, most of them (close to 60%) can only operate the device three times, and 98% of them can only operate the device ten times before they are identified and logged out (Figure 14b). The average value is only 4.443 operations, which means the attackers can only operate the device for 4.23 s on average before being detected by BioTouch. In summary, BioTouch is a secure and usage transparent scheme for user re-authentication. It has high resistance to attackers and ensures a good user experience for legitimate users.

### 8.8. System Power Consumption

**Figure 15:**
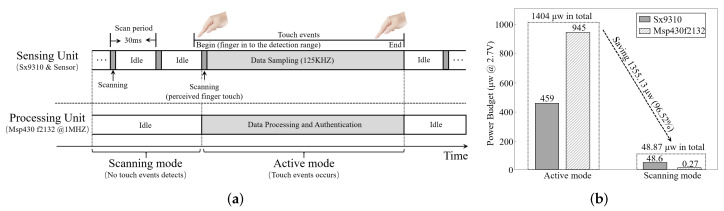
When the user’s finger enters the sensing range (2.5 cm in the test), BioTouch samples the sensed bio-capacitance and processes the data to authenticate the user. If the finger is out of range, BioTouch shuts down the processing unit and only scans the sensor periodically to further reduce power consumption. (**a**) System operating mode. (**b**) System power consumption.

Finally, we evaluate the power consumption of BioTouch. To reduce the power consumption and extend the battery life, we set an event-triggered mode in BioTouch and carried out a lightweight system design. When a touch event occurs, BioTouch samples the sensor at 125 kHz and starts user identification, which consumes 1.404 mW in total. When the system stands by and waits for touching events (e.g., the user is watching a movie), BioTouch periodically scans the sensor at 33 Hz (30 ms scan period) and turns off all extra components and consumes only 48.87 μW in total. The operating modes and corresponding power consumption of BioTouch are shown in Figure 15a,b, respectively. Statistics show that the average mobile device usage time per person worldwide is 3.25 h per day [56]. If the total duration of the touch operation lasts two hours per day, BioTouch only consumes 0.022% of the device’s battery power (the average battery capacity of current mobile devices is approximately 12,950 mWh (3500 mAh@3.7 V) [57]).

## 9. Discussion

The main external influence, future work and ethical concerns of BioTouch are discussed as follows:

### 9.1. External Influence

**Figure 16:**
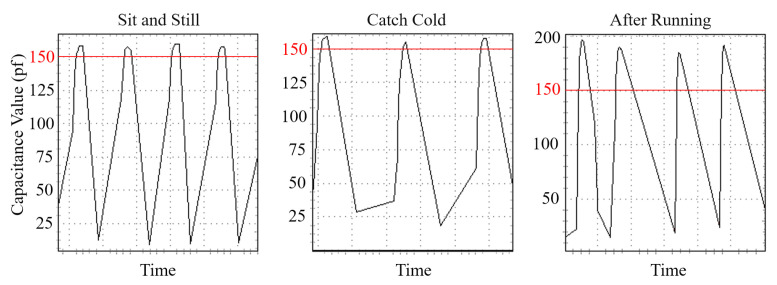
Equivalent capacitance of finger-touching operations under different body conditions. It can be seen from the figure that the user’s finger-touching operation is relatively stable when the user is sitting and still (**left**). When the user has a cold, their behavior changes due to weak body conditions, but the bio-capacitance (peak value) remains stable (**middle**). After strenuous exercise, the sensed bio-capacitance changes dramatically as the sweat changes the body conductivity (**right**).

We considered external influences from the impact of the user’s physical condition, wet fingers and static electricity carried on the finger that may influence system performance as follows:

- User body condition. We consider that if a user becomes ill or after strenuous exercise, their behavior might be affected by body conditions, which affects the accuracy of re-authentication. Indeed, such changes might vary from person to person, which is hard to predict. The sensed bio-capacitances of a user under different body conditions are shown in Figure 16. We could tell that the user’s characteristics change in different states. Therefore, the limitation of BioTouch is that it cannot cover those special application scenarios.
- Wet finger. We also considered possible performance influence on wet fingers. We asked ten users to wet their fingers and operate the device. The result shows that all users (including the device owner) are identified as attackers and logged out by the system. The reason stems from the fact that the sensed capacitance value changes much more than different users as the wet finger wets the sensor during touching. Under such conditions, neither the owner nor attackers can fit the information with the stored legitimate data set. Despite the fact that the system does not work well in terms of influencing the usage of the owner, it does not bring security risks of falsely accepting attackers.
- Static Electricity. Finally, we considered the possible effect of static electricity on our system, which may occur in a dry environment such as in the winter. Through the test, we found that the static electricity could only affect the first touch as it discharges and will not affect all subsequent operations. In addition, we also added a Zener diode in the prototype circuit, which could effectively protect the system hardware from static electricity discharges.

### 9.2. Future Works

To combat the influence from different user body conditions, as a solution, we may use the other information to identify the user’s current physical condition and calibrate the system parameters accordingly. That user information can be collected when the user unlocks the device and logs in. For example, if the user uses face recognition to unlock the device, we may identify whether the user is sick or not based on the user’s skin color. Moreover, we may use built-in accelerometers to collect vibrations to identify whether the user is currently stationary or in motion.

We also consider the possible solution to enhance the system robustness in worse scenarios. For example, to combat the influence of wet finger, a sensor array might be useful. Specifically, we can utilize sensor array to capture the user’s touch operation in multiple dimensions. Despite wet fingers influencing each sensor and affecting the results, it is possible to eliminate the interference by data differential base on multiple sensors. At the same time, mass data collection and learning on wet finger touching might also be very useful; we will try that in future works.

### 9.3. Technical Specifications Comparison

At the end of this paper, we would like to discuss the technical specifications of our system and the state-of-art related works. We selected six representative works and made a comparison with our design, as presented in Table 4.

We can find that our work has the highest accuracy and can detect attackers in the fewest operations. This achievement benefits from our user legitimacy model (Section 6.2), which takes the authentication results of multiple touching operations into account, thus effectively eliminating misjudgments and significantly improving the accuracy. Besides, as our system works based on normal touching operations, it can cover the entire usage on touching devices. In addition, our design is also transparent to users in using as it requires no user assistance like wearing additional hardware, facing the camera and walking. Benefitting with above merits, we believe that BioTouch is could possibly be widely deployed in the future, bringing continuous security insurance for capacitive touching devices in usage.

### 9.4. Ethical Concerns

In this paper, the collected user data are only used and stored locally, without appearing on the Internet. Thus, we believe that our work does not involve ethical issues and user privacy leakage.

## 10. Conclusions

In this paper, we propose BioTouch, a novel method to re-authenticate users during device usage. BioTouch collects both user behavior and biometric information to achieve accurate re-authentication during touching operations. This design features both secure and usage transparency. To achieve our design, we developed a user-legitimacy model to comprehensively describe the user’s legitimacy based on the identification results of multiple touching operations. The experimental results show that BioTouch can identify 98% of attackers within 10 s and achieves up to 99.84% accuracy during usage. For legitimate users, the misjudgment is only 0.321% in 16 min usage and 0.89% in 2.6 h of long-time usage. Besides, BioTouch is a lightweight design that does not require intensive computations and power (1.4 mW in peak). With the merits of continuous security, transparency in use and low power consumption, we foresee that BioTouch is expected to be widely deployed in the future.

## Figures and Tables

**Table 1:** Summary of related works on user re-authentication.

Feature/Approach	Required User Cooperation	FAR ${}^{1}$	Robustness
Facial [10]	Face to camera	0.5%	✓
Voice [13]	Talk	0.1%	✓
Gait [14,15,16]	Walk	2.13%	✓
Mouse and Keyboard Operation [17,18,19,20]	None	4.75%	×
Touching Track [21]	None	4.32%	×
Vibration [22]	None	3.3%	×
BioTouch (This Work)	None	0.32%	✓

^1^ False-Alarm-Rate. We show the mean value if multiple works are listed.

**Table 2:** The parameter configuration in the system prototype.

Parameter	Value	Description
Scan Period	30 ms	The interval to scan touch events in system standby (as described in Section 8.8)
Sampling Frequency	125 kHz	The frequency to sample the sensor when a touching event is detected.
Filter Threshold	16 Samples	The system filters the sampled result in every 16 consecutive samples (take the average value).
Resolution	0.015 pF	The set resolution for the prototype to sense the bio-capacitance during user touching.
Signal Gain	32 times	The collected signal is gained by 32 times to make a better distinguish between different users.
Sensing Range	240 pF	The capacitance sensing range is up to 240 pF (7.5 pF× 32) with gain.

**Table 3:** Motion Detect Rate (We select 10 users from 50 users as follows).

User ID	Gender	Environment Temperature	Motion	Actual Motions	Detected Motions	Missed Detection
1	Male	30 ${}^{\circ }$C	Tapping	68	68	0
6	Female	30 ${}^{\circ }$C	Sliding	57	56	1
10	Female	10 ${}^{\circ }$C	Holding	49	49	0
12	Male	30 ${}^{\circ }$C	Tapping	93	92	1
20	Male	10 ${}^{\circ }$C	Holding	41	41	0
25	Female	10 ${}^{\circ }$C	Tapping	61	59	2
32	Female	30 ${}^{\circ }$C	Sliding	96	91	1
38	Male	30 ${}^{\circ }$C	Tapping	71	71	0
43	Female	10 ${}^{\circ }$C	Holding	56	56	0
47	Male	10 ${}^{\circ }$C	Sliding	64	64	0
Motion detection rate among 50 users: 98.9%.						

**Table 4:** Technical specifications and application requirements comparison with different re-authentication schemes.

Scheme	Technical Specifications	Application Requirements
Facial-Based [10]	Can logout 73 of the 82 attackers (89%) in 2 min.	Required users keep facing the front camera during use and avoid obstruction.
Voice-Based [13]	Achieve 97% accuracy with 30 voice commands.	Require voice assistance, hence does not suit quiet environments and long-time usage.
Gait-Based [16]	Achieve 98.5% accuracy with acceleration, angular velocity and PPG ${}^{1}$ information in walking.	Require users to wear customized equipment for information collection, and performance well only in walking state.
Keystroke and mouse movement based [17]	Logout attackers on average after 252 actions.	Can be applied to computers that operate with the mouse and keyboard.
Touching track based [21]	Achieves 79% to 95% accuracy in a variety of different touching gestures.	Works base on finger movement trajectory, suitable for touching devices.
Vibration [22]	Achieve 96.7% accuracy in 20 s of usage.	Work based device vibration in using, suit for device handheld scenarios.
BioTouch (Our Design)	Logout attackers on average after 4.43 operations and achieves 99.84% accuracy after 18 operations.	Work base on touching operations and keep transparent in the entire touching usage.

^1^ PPG: the photoplethysmography signal, which indicates the blood volume changes in the microvascular bed of tissue.

## Data Availability

The data presented in this study are available on request from the first author.

## Abbreviations

The following abbreviations are used in this manuscript:

HMI	Human Machine Interface
CDF	Cumulative Distribution Function
SVM	Support Vector Machine
FAR	False Alarm Rate
FPR	False Positive Rate
AFE	Analog-Front-End
ADC	Analogue to Digital Converter
PPG	Photoplethysmography
